# The Evaluation of the Reactivating and Neuroprotective Efficacy of Two Newly Prepared Bispyridinium Oximes (K305, K307) in Tabun-Poisoned Rats—A Comparison with Trimedoxime and the Oxime K203

**DOI:** 10.3390/molecules22071152

**Published:** 2017-07-11

**Authors:** Jiri Kassa, Jan Misik, Jana Hatlapatkova, Jana Zdarova Karasova, Vendula Sepsova, Filip Caisberger, Jaroslav Pejchal

**Affiliations:** 1 Department of Toxicology and Military Pharmacy, Faculty of Military Health Sciences, University of Defense, Trebesska 1575, 500 01 Hradec Kralove, Czech Republic; misik@pmfhk.cz (J.M.); hatlapatkova.j@seznam.cz (J.H.); zdarova.jana@gmail.com (J.Z.K.); sepsova@pmfhk.cz (V.S.); CaisbergerF@lfhk.cuni.cz (F.C.); jaroslav.pejchal@unob.cz (J.P.); 2 Clinic of Neurology, Faculty Hospital Hradec Kralove, Sokolovska 581, 500 01 Hradec Kralove, Czech Republic

**Keywords:** tabun, acetylcholinesterase, neurotoxicity, functional observational battery, histopathology, oximes, rats

## Abstract

The ability of two newly developed oximes (K305, K307) to protect tabun-poisoned rats from tabun-induced inhibition of brain acetylcholinesterase, acute neurotoxic signs and symptoms and brain damage was compared with that of the oxime K203 and trimedoxime. The reactivating and neuroprotective effects of the oximes studied combined with atropine on rats poisoned with tabun at a sublethal dose were evaluated. The reactivating efficacy of a newly developed oxime K305 is lower compared to the reactivating efficacy of the oxime K203 and trimedoxime while the ability of the oxime K307 to reactivate tabun-inhibited acetylcholinesterase (AChE) in the brain roughly corresponds to the reactivating efficacy of the oxime K203 and it is slightly lower compared to trimedoxime. In addition, only one newly developed oxime (K307) combined with atropine was able to markedly decrease tabun-induced neurotoxicity although it did not eliminate all tabun-induced acute neurotoxic signs and symptoms. These results correspond to the histopathological evaluation of tabun-induced brain damage. Therefore, the newly developed oximes are not suitable for the replacement of commonly used oximes (especially trimedoxime) in the treatment of acute tabun poisonings.

## 1. Introduction

Highly toxic organophosphorus compounds have been developed as chemical warfare agents called nerve agents. They are considered to be the most dangerous chemical warfare agents because of their high toxicity, rapid onset of clinical signs and symptoms and rapid progression of acute poisoning to death. Their acute toxic effects are based on the phosphonylation of acetylcholinesterase (AChE, Enzyme Comission (EC) 3.1.1.7), the enzyme responsible for hydrolysis of neurotransmitter acetylcholine in muscular and neural synaptic clefts, leading to the irreversible inhibition of its active site and subsequent overstimulation of postsynaptic cholinergic receptors due to the accumulation of the neurotransmitter acetylcholine in synapses of the central and peripheral nervous systems. The overstimulation of cholinergic receptors results in muscarinic and nicotinic signs and symptoms including excitotoxicity, seizures and brain damage. The death is usually caused by respiratory failure resulting from bronchospasm, excessive bronchial secretion, paralysis of respiratory muscles, and depression of brain respiratory centers [1,2].

The medical countermeasures of nerve agent poisonings include the administration of the antidotes that are able to counteract the main acute toxic effects of nerve agents. A current standard antidotal treatment usually represents a combined administration of an anticholinergic drug (preferably atropine) and an oxime (preferably pralidoxime or obidoxime). Generally, anticholinergic drugs are used for relieving muscarinic signs and symptoms whereas oximes are used for the reactivation of nerve agent-inhibited AChE by dephosphonylating the enzyme active site and restoring its activity [1,3]. While a lot of these reactivators are sufficiently effective to reactivate sarin or VX agent-inhibited AChE, their ability to reactivate soman, cyclosarin or tabun-inhibited AChE is generally low [4,5].

One of the most resistant nerve agents is tabun (*O*-ethyl-*N,N*-dimethyl phosphoramido-cyanidate). It differs from other highly toxic organophosphates in its chemical structure and by the fact that commonly used antidotes are not able to sufficiently prevent tabun-induced acute toxic effects. The toxic effects of tabun are problematically antagonized due to the changes in hydrogen bonding and the conformational changes of AChE-tabun complex prior to an aging process in the AChE active site [6,7].

In the case of severe intoxication, some nerve agents including tabun can cause centrally mediated seizure activity that can rapidly progress to status epilepticus and contribute to profound brain damage that is associated with long-lasting neurological and psychological injuries [8,9]. Therefore, the ability of antidotes to reactivate nerve agent-inhibited brain AChE, counteract acute neurotoxic effects of nerve agents and prevent nerve agent-poisoned organisms from irreversible lesions in the central nervous system (CNS) is very important for the successful antidotal treatment of acute nerve agent poisonings. Generally, the oximes exert more potent effects in the peripheral nervous system compared to CNS due to their low penetration across the blood-brain barrier (BBB) although the penetration of oximes into CNS and subsequent reactivation of nerve agent-inhibited AChE in the brain was previously described [10,11,12].

The commonly used monopyridinium (e.g., pralidoxime) and bispyridinium oximes (e.g., obidoxime, trimedoxime, HI-6) are not able to sufficiently counteract the acute toxic effects of tabun because of their low ability to reactivate tabun-inhibited AChE [4,13,14]. Therefore, the replacement of commonly used oximes (pralidoxime, obidoxime, HI-6) with a more effective oxime has been a long-standing goal for the treatment of tabun poisoning [15].

**Figure 1:**
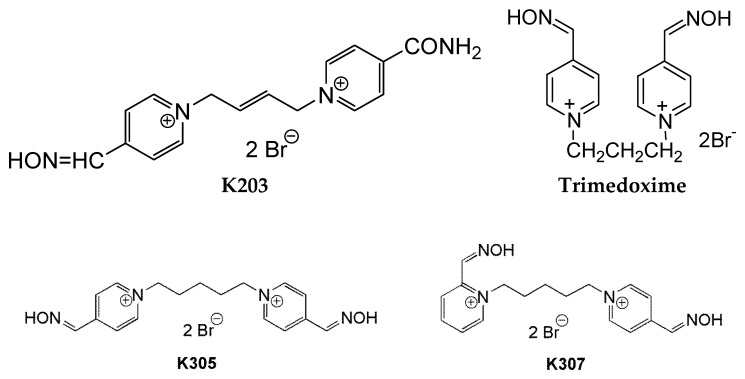
Chemical structures of the studied oximes.

Among recently developed oximes, the oxime K203 has been considered to be promising reactivator of tabun-inhibited AChE. However, the differences between the reactivating and therapeutic efficacy of the oxime K203 and commonly used bispyridinium oximes (obidoxime, trimedoxime) are not significant [16]. Therefore, we are still searching for a more efficacious oxime able to sufficiently reactivate tabun-inhibited AChE. For this purpose, two novel oximes, K305 [1,5-bis(4-hydroxyiminomethylpyridinium)-pentane dibromide] and K307 (1-[2-(hydroxyiminomethyl) pyridinium-1-yl)-pent-1-yl]-4-(hydroxyiminomethyl) pyridinium dibromide) (Figure 1), were synthesized at our Department of Toxicology and Military Pharmacy to improve the efficacy of antidotal treatment in reactivating tabun-inhibited AChE and eliminating tabun-induced acute toxicity. They were developed based on the structure-activity relationship study and they were chosen based on the data obtained from in vitro evaluation of their ability to reactivate tabun-inhibited AChE [17]. The aim of this study was to compare the reactivating and neuroprotective efficacy of both newly developed oximes (K305, K307) with the oxime K203 and trimedoxime in combination with an anticholinergic drug atropine in tabun-poisoned rats. The tabun-induced neurotoxic signs were determined using a functional observational battery (FOB), a non-invasive and relatively sensitive type of neurological examination for a wide range of neurobiological functions including measurements of sensory, motor and autonomic nervous functions. The tabun-induced brain damage was investigated by the histopathological evaluation using hematoxylin-eosin and Fluoro-Jade^®^ C staining.

## 2. Results

### 2.1. The Evaluation of Neuprotective Efficacy of the Studied Oximes in Tabun-Poisoned Rats

The results of the experiments related to the measurement of tabun-induced neurotoxicity at 2 h following tabun poisoning are summarized in Table 1. While five non-treated tabun-poisoned rats, two tabun-poisoned rats treated with the oxime K305 and atropine and one tabun-poisoned rat treated with the oxime K203 and atropine died before the evaluation of tabun-induced neurotoxicity by FOB, all tabun-poisoned rats treated with atropine in combination with trimedoxime or the oxime K307 survived till the end of experiment. The evaluation of tabun-induced neurotoxic signs at 2 h following intoxication proved significant alteration of 36 observed parameters. Tabun produced passive behavior of rats during handling and retention, miosis, marked salivation and nose secretion and a decrease in muscular tone. The posture of tabun-poisoned rats was seriously altered and fur as well as skin abnormalities were observed. Exploratory and rearing activities were significantly reduced, righting reflex was altered, strong tremors and hyperkinesis were observed, gait and mobility were seriously impaired and ataxia was found. In addition, no reaction during a reflex testing consisting of recording each rat’s response to the frontal approach of the blunt end of a pen, to the touch of the pen to the posterior flank, to an auditory click stimulus and to a pinch on the tail was found. The pupils of tabun-poisoned rats did not constrict in response to light because of tabun-induced miosis. A significant decrease in landing foot splay, forelimb and hindlimb grip strength and vertical as well as horizontal activity was also observed at 2 h following tabun challenge (Table 1). Only one of newly developed oximes (K307) in combination with atropine was able to prevent some tabun-induced signs of neurotoxicity observed at 2 h following tabun challenge with the exception of miosis, a decrease in rearing activity, alteration of righting reflex, slight impairment of gait, the absence of pupil response to light, a decrease in forelimb and hindlimb grip strength and vertical activity (Table 1). Its ability to protect tabun-poisoned rats from tabun-induced acute neurotoxicity roughly corresponds to the oxime K203. On the other hand, the ability of trimedoxime to eliminate or at least reduce tabun-induced signs of acute neurotoxicity was slightly higher compared to the oxime K307. The miosis, the absence of pupil response to light, alteration of righting reflex and a decrease in hindlimb grip strength and vertical activity were only observed in the case of treatment of tabun-poisoned rats with trimedoxime (Table 1).

The results of the experiments related to the measurement of tabun-induced neurotoxicity at 24 h following tabun poisoning are summarized in Table 2. Five non-treated tabun-poisoned rats, four tabun-poisoned rats treated with the oxime K305 and atropine and two tabun-poisoned rats treated with the oxime K203, K307 or trimedoxime in combination with atropine died before the evaluation of tabun-induced neurotoxicity by FOB. The evaluation of tabun-induced neurotoxic signs at 24 h following intoxication proved significant alteration of practically all observed parameters with the exception of urination and defecation (Table 2). Only one of newly developed oximes (K307) in combination with atropine was able to prevent many tabun-induced signs of neurotoxicity observed at 24 h following tabun challenge with the exception of handling of rats, slight nose secretion, the absence of tail-pinch response and a decrease in landing foot splay, hindlimb grip strength and vertical as well as horizontal activity (Table 2). On the other hand, the ability of trimedoxime and the oxime K203 to eliminate or at least reduce tabun-induced signs of acute neurotoxicity was slightly higher compared to the oxime K307. They were not be able to eliminate alteration of righting reflex and a decrease in landing foot splay, hindlimb grip strength and vertical as well as horizontal activity (Table 2).

### 2.2. The Evaluation of Reactivating Efficacy of the Studied Oximes in the Brain of Tabun-Poisoned Rats

The ability of studied oximes to eliminate or reduce tabun-induced signs and symptoms of neurotoxicity corresponds to their ability to reactivate tabun-inhibited AChE in the brain. While the reactivating efficacy of the oxime K305 was wery low (less than 5%), another newly developed oxime K307 was able to increase the activity of tabun-inhibited AChE by more than 10%. The highest reactivating efficacy was shown for trimedoxime (almost 14%), while the ability of the oxime K203 to reactivate tabun-inhibited AChE in the brain was slightly lower compared to the oxime K307 (less than 9%) (Table 3).

The differences among tested oximes are not significant, however, only trimedoxime and the oxime K307 reactivating efficacy was higher than minimal reactivation activity (10%) that is considered to be needed for saving the life of an intoxicated organism [18]. This result is supported by the fact that all tabun-poisoned rats survived at 2 h after tabun challenge only in the groups of rats treated with trimedoxime or the oxime K307.

### 2.3. Histopathological Evaluation of Tabun-Induced Brain Damage

The most extensive alterations were found in tabun-poisoned animals without antidotal treatment. In this group, significantly increased histopathological damage scores were found in cerebellum, cortex, hippocampus, piriform cortex and thalamus (*p* = 0.009, 0.005, 0.026, 0.031 and 0.022, respectively) when compared to control (Table 4). Diffuse necrotic changes in amygdaloid body and thalamus were present in two of three animals, while cortex and piriform cortex displayed pseudolaminar pattern of damage in three and two cases, respectively. In the remaining areas, focal degenerative/necrotic changes accompanied with oedema were present. Especially, basolateral nucleus of amygdaloid body, the third, fourth and fifth layer of cortex, neurons in dentate gyrus (>CA1 > CA3 > CA2 zone) of hippocampus, perifornical hypothalamic nuclei, the second layer of piriform cortex, and medial, ventromedial and ventrolateral thalamic nuclei were markedly impaired. Hemorrhage was not present. Fluoro-Jade^®^ C positivity increased in all evaluated areas, however, since only three animals survived until the time of examination, statistical significance was only calculated in amygdaloid body, cortex and piriform cortex (0.026, 0.031 and 0.025, respectively) (Table 5). Interestingly, out of scoring system, Fluoro-Jade^®^ C positive cells were found in zona incerta of two affected animals. None of newly developed oximes (K305 and K307) administered with atropine was able to significantly improve histopathological findings or Fluoro-Jade^®^ C positivity in comparison with tabun-poisoned rats. In K305 and K307 group, three of four and one of six animals, respectively, displayed diffuse degenerative/necrotic changes in at least 1 selected brain area. Strong Fluoro-Jade^®^ C positivity was found in zona incerta of two animals in the K305 group. K203 and atropine administration significantly reduced tabun-induced histopathological damage in cortex (*p* = 0.048) (Table 4), nevertheless, diffuse Fluoro-Jade^®^ C positivity in one of six animals was found. The lowest extent of damage was observed in trimedoxime and atropine-treated group. This treatment significantly reduced histopathological score in amygdaloid body, cerebellum, cortex, piriform cortex and thalamus (*p* = 0.049, 0.015, 0.009, 0.020, and 0.034, respectively) (Table 4) and Fluoro-Jade^®^ C positivity in amygdaloid body (*p* = 0.040) (Table 5). Diffuse vasogenic oedema was found in one of six animals in this group.

## 3. Discussion

The repeated use of nerve agents against military forces and civilian populations, in particular the recent attacks with sarin in Syria, emphasizes the need of sufficiently effective antidotal treatment [19,20]. The severe poisoning with nerve agents including tabun brings centrally mediated seizures. These seizures can rapidly progress to status epilepticus and cause irreversible seizure-related brain damage if left untreated [21,22,23]. Nerve agent-induced long-lasting hyperstimulation of central cholinergic muscarinic receptors leads to a burst of excitatory animo acids including glutamate which stimulates the α-amino-3-hydroxy-5-methyl-4-isoxazolepropionic acid (AMPA) and *N*-methyl-d-aspartate (NMDA) glutamate receptors [23,24]. Thus, sustained seizures (status epilepticus) are probably associated with increased glutamatergic activity leading to excitotoxic damage predominantly in the hippocampus, amygdala, piriform and entorhinal cortices [23]. Therefore, the ability of antidotes to counteract acute neurotoxic effects of nerve agents is important for the successful antidotal treatment of acute nerve agent poisonings.

According to previously published data, atropine alone fails to prevent nerve agent-induced acute neurotoxic effects following an exposure to nerve agents at sublethal doses [25] because it is considered to be a muscarinic blocker with a relatively low central antimuscarinic activity in comparison with other anticholinergic agents such as benactyzine, biperiden and scopolamine [26]. As the potential benefit of atropine alone to counteract the acute neurotoxicity of nerve agents is negligible, atropine should be combined with AChE reactivator for the antidotal treatment of nerve agent poisonings to improve its neuroprotective efficacy.

Oximes are not equally effective against all available nerve agents. Their effectiveness depends on many factors, especially on the chemical structure of nerve agents and the rate of ageing of enzyme-inhibitor complex because the aged enzyme cannot be reactivated. The ageing kinetics of different nerve agents is different, ranging from a few minutes to many hours [27]. Other factors that influence oxime therapy include inhibition potency of nerve agent, its toxicokinetics, reactivating potency of oxime and its pharmacokinetics, correct dosing, evaluation for the persistent need of oxime therapy and correct timing [27,28]. Generally, the ability of currently available oximes to eliminate tabun-induced acute neurotoxic effects is relatively low [29]. Among them, trimedoxime seems to be the most effective to counteract tabun-induced acute neurotoxicity in rats, although it is not able to completely eliminate tabun-induced signs of neurotoxicity in the case of sublethal tabun poisoning, either [25]. The evaluation of the neuroprotective efficacy of the oxime K203 in tabun-poisoned rats brought relatively promising results but the differences between neuroprotective efficacy of the oxime K203 and commonly used oximes are not so high [30]. The unsatisfactory efficacy of currently available and recently developed oximes in eliminating tabun-induced acute neurotoxicity can be explained by low potency of oximes in reactivating tabun-inhibited AChE in vitro and in vivo [1,4,13,31] and limited penetration across BBB [1,32]. Therefore, new oximes with higher potency to reactivate tabun-inhibited AChE and counteract tabun-induced acute neurotoxicity are still sought to increase the efficacy of antidotal treatment of acute tabun poisonings.

Bispyridinium oximes are generally more effective to reactivate nerve agent-inhibited AChE than monopyridinium oximes, however, their ability to penetrate across BBB is lower, maximally 6% [12]. A design of newly developed oximes was based on the data obtained during the extensive work on oxime development and from structure-activity relationship studies realized at our Department of Toxicology and Military Pharmacy [33,34,35].

Our results demonstrate that the potency of studied oximes to eliminate or at least reduce tabun-induced neurotoxic signs and symptoms is similar but not the same. To compare the neuroprotective efficacy of newly developed oximes (K305, K307) with trimedoxime and the oxime K203, the ability of one novel oxime (K307) to eliminate tabun-induced neurotoxic signs and symptoms was slightly lower than the neuroprotective efficacy of trimedoxime while the ability of another novel oxime K305 to eliminate or at least reduce acute neurotoxic signs and symptoms of tabun was negligible. The differences in the neuroprotective efficacy of studied oximes roughly correspond to their reactivating efficacy in the brain because the ability of the oxime K307 to reactivate tabun-inhibited AChE in the brain was markedly higher compared to another novel oxime K305 but slightly lower compared to trimedoxime that is considered to be one of the most efficient reactivators in tabun poisoning. However, its potency to reactivate tabun-inhibited AChE and eliminate tabun-induced neurotoxic effects is also limited when it is administered at low, human relevant doses [4,13,36,37]. Additionally, these results corresponded to the histopathological evaluation demostrating trimedoxime to be the most effective reactivator of tabun-inhibited AChE (>K203 ~ K307 > K305) However, even in trimedoxime and atropine-treated group, tabun-poisoned animals suffered from mild neural damage, which tends to progress over time [38]. Thus, the benefit of both novel bispyridinium oximes for neuroprotective efficacy of antidotal treatment of acute tabun poisonings is not so high to make the decision about the replacement of commonly used oximes (especially trimedoxime) in the antidotal treatment of acute tabun poisonings. Nevertheless, it is necessary to mention that the strength of these results is limited because five non-treated tabun-poisoned rats died before the evaluation of tabun-induced neurotoxicity by FOB.

## 4. Materials and Methods

### 4.1. Animals

Male albino Wistar rats weighing 220–250 g were purchased from VELAZ (Prague, Czech Republic). They were kept in an air-conditioned room (22 ± 2 °C and 50 ± 10% relative humidity, with lights from 7.00 to 19.00 h) and allowed access to standard food and tap water *ad libitum*. The rats were divided into groups of 8 animals. Handling of the experimental animals was done under the supervision of the Ethics Committee of the Faculty of Military Health Sciences, Czech Republic.

### 4.2. Chemicals

Tabun was obtained from the Technical Institute in Brno (Czech Republic) and was 92% pure. Its purity was assayed by acidimetric titration. The basic solution of tabun (1 mg/1 mL) was prepared in propyleneglycol three days before starting the experiments. Actual solution of tabun was prepared from its basic solution with the help of saline immediately before its administration. All oximes (K305, K307, K203, trimedoxime) were synthesized at our Department of Toxicology and Military Pharmacy of the Faculty of Military Health Sciences (Czech Republic). Their purity was analyzed using HPLC technique with UV detection (310 nm) and they were more than 96% pure [39]. All other drugs and chemicals of analytical grade were obtained commercially (Sigma-Aldrich, Prague, Czech Republic) and used without further purification. The saline solution (0.9% NaCl) was used as a vehicle. All substances were administered intramuscularly (i.m.) at a volume of 1 mL/kg body weight (b.w.).

### 4.3. In Vivo Experiments

Tabun was administered at a sublethal dose (150 µg/kg b.w.—85% LD50). One minute following tabun poisoning, the rats were treated with atropine (10 mg/kg b.w.) in combination with the oxime K305, K307, K203 or trimedoxime at equitoxic doses corresponding to 5% of their LD50 values [40]. Totally, six groups of animals were used in this study—control group (rats were administered with saline instead of tabun and antidotes), tabun group (tabun poisoning without antidotes), tabun + atropine + K203 group (tabun poisoning treated with atropine and the oxime K203), tabun + atropine + trimedoxime group (tabun poisoning treated with atropine and trimedoxime), tabun + atropine + K305 group (tabun poisoning treated with atropine and the oxime K305) and tabun + atropine + K307 group (tabun poisoning treated with atropine and the oxime K307). The neurotoxicity of tabun was monitored using FOB at 2 and 24 h following tabun poisoning. The evaluated markers of tabun-induced neurotoxicity in experimental animals were compared with the parameters obtained from control rats given saline instead of tabun and antidotes at the same volume (1 mL/kg b.w.). FOB consists of 42 measurements of sensory, motor and autonomic nervous functions. Some of them are scored (Table 6), the others are measured in absolute units [41]. The first evaluation was obtained when tabun-poisoned rats were in the home cage. The observer evaluated each animal’s posture, palpebral closure and involuntary motor movements. Then, each rat was removed from the home cage and briefly hand-held. The exploratory activity, piloerection and other skin abnormalities were noted. Salivation and nose secretion were also registered and scored. Subsequently, the rats were placed on a flat surface which served as an open field. A timer was started for 3 min during which the frequency of rearing responses was recorded. At the same time, gait characteristics were noted and ranked, and stereotypy and bizarre behaviors and abnormal posture were evaluated. At the end of the third minute, the number of fecal boluses and urine pools on the adsorbent pad was registered. Reflex testing comprising recording each rat’s response to a frontal approach of the blunt end of a pen, a touch of the pen to the posterior flank and to an auditory click stimulus was also used.

The response to a pinch on the tail and the ability of pupils to constrict in response to light were then assessed. These measures were followed by a test for the aerial righting reflex and by the measurements of forelimb and hindlimb grip strength and finally hindlimb landing foot splay. Motor activity data were collected by means of an apparatus for testing spontaneous motor activity of laboratory animals (constructed at the Faculty of Military Health Sciences, Hradec Kralove, Czech Republic) with the same animals and under the same conditions. The apparatus represents the plastic cage (168 × 55 × 21 cm) divided in four separate parts (four animals can be measured at the same time). Each part of the cage contains two infrared detectors equipped with a Fresnel lens—the lower located detector monitors all horizontal movements, while the higher located detector monitors all vertical movements of the experimental animal. The rat was placed for a short period of time (10 min) in the cage and all vertical and horizontal movements were monitored. Signals produced by detectors were transformed into computer signals with the help of optoelectronic interface and then analysed. The observer was blind to the treatment condition. Data collected with the FOB include categorial, ordinal and continuous values. Their statistical analyses were performed on a PC with a special interactive programme NTX. The categorial and ordinal values were formulated as contingency tables and judged consecutively by Chi-squared test of homogeneity, Concordance-Discordance test and Kruskal-Wallis test, respectively. The continual data were assessed by successive statistical tests: CI for Delta, Barlett test for Equality of Variance, Williams test and Test for Distribution Functions. The results of experimental groups were compared to the condition of the control rats. The differences were evaluated at the significant level *p* < 0.05. To evaluate the reactivating efficacy of the oximes in the brain, the rats were decapitated after FOB test (24 h 30 min after intoxication), totally exsanguinated and the brain was rapidly removed, cut in half along the midsagittal plane and one half of the brain was immediately frozen at the temperature −70 °C. Within three days, they were homogenized in Tris-HCl buffer (0.02 mol/L, pH 7.6, 1:10) to determine AChE activity by standard spectrophotometric method [42]. The AChE activity was measured in individual brains. Acetylthiocholine was used as a substrate (Tris-HCl buffer, N = 0.1 mol/L, pH 7.6). Helios Alpha, the spectrophotometer was used for determination of absorbancy at 436 nm. The AChE activity was expressed as µkat/kg (µmol substrate hydrolyzed/kg wet tissue within 1 s). The untreated control values of brain AChE activity were obtained from rats administered with saline buffer (physiological solution—0.9% NaCl) instead of tabun and antidotes (saline control). The percentage of reactivation was calculated using the AChE activity values: {1 − [((controls) − (tabun + oxime + atropine))/((controls) − (tabun group))]} × 100 [43]. All experiments were perfomed in the same part of the day (from 08:00 h to 10.00 h). The variability was statistically evaluated by the standard deviation (SD) calculated for each group. The differences between groups were calculated using means ± SD and the statistical significance was tested by one-way ANOVA test with Scheffe’s post hoc test. The differences were considered significant when *p* < 0.05.

To evaluate histopathological changes after tabun poisoning, second half of brain was fixed with a 10% neutral buffered formalin (Chemapol, Prague, Czech Republic). Samples were subsequently embedded into paraffin (Paramix, Holice, Czech Republic) and 6 µm thick coronary brain sections (four sections for each staining method) were cut (Microtome model SM2000 R, Leica, Wetzlar, Germany) at level between 3 mm and 4.2 mm (to evaluate all structures with exception of cerebellum) and between 11.2 mm and 12.0 mm (to evaluate cerebellum) from bregma according to Paxinos stereotactic brain atlas [44]. For hematoxylin-eosin staining, selected brain sections were dewaxed and rehydrated through xylene and an alcohol series (all from Kulich, Hradec Kralove, Czech Republic) and stained with hematoxylin and eosin (both Merck, Prague, Czech Republic). The histological changes were scored using a BX-51 microscope (Olympus, Prague, Czech Republic) and following semi-quantitative criteria: 0—no pathology, 1—more than 3 neurons with nucleus margination, chromatolysis, vacuolar degeneration and/or shrunken eosinophilic neurons in 1 nucleus/subregion, 2—shrunken eosinophilic neurons in more than 1 nucleus/subregion, 3—focal damage or multiple changes described in (2) and present in ≥2 nucleus/subregions and/or hemorrhage, and 4—diffuse damage of the region and/or multiple hemorrhages. For Fluoro-Jade^®^ C staining, paraffin sections were dewaxed and rehydrated through xylene and an alcohol series. Afterwards, the sections were immersed in 0.06% potassium permanganate (Sigma-Aldrich) for 10 min, rinsed in distilled water for 2 min and dyed by 0.0002% solution of the Fluoro-Jade^®^ C (Merck, Prague, Czech Republic) for 20 min in dark. Washed (3 distilled water washes, 1 min each) and dried slides were mounted with DPX (Sigma-Aldrich) in dark. Fluorescent cells were evaluated using a BX-51 microscope (Olympus) with green excitation fluorescence filter and following semi-quantitative criteria: 0—no fluorescent cells, 1—1–2 fluorescent cells in 1 nucleus/subregion, 2—≥ 3 fluorescent cells in 1 nucleus/subregion or 1–2 fluorescent cells in ≥2 nucleus/subregions, 3—multiple fluorescent cells in ≥2 nucleus/subregions and/or hemorrhage, 4—diffuse fluorescent positivity and/or multiple hemorrhages. The Kruskal-Wallis test with subsequent multiple pairwise comparisons (IBM SPSS Statistics Version 22.0 software, IBM Corp., Armonk, NY, USA) was used to evaluate differences between all groups within particular brain nucleus/subregion. The differences were considered significant when *p* ≤ 0.05.

## 5. Conclusions

The changes of the structure of commonly used oximes realized according to the postulated requirements [34] are not able to markedly increase the potency of current antidotal treatment to eliminate tabun-induced acute neurotoxicity, probably due to conformational changes of AChE-tabun complex in AChE active site that make the nucleophilic attack of oximes very difficult [6,7] and due to low penetration of these oximes across BBB [32]. Thus, it is necessary to find a new approach how to change the known structures of AChE reactivators to reach better entering into the active site of AChE-tabun complex and higher penetration through BBB. The higher brain concentration of AChE reactivator should bring higher reactivation of nerve agent-inhibited brain AChE and more effective elimination of acute neurotoxic signs and symptoms of nerve agents including tabun.

## Figures and Tables

**Table 1:** The values of tabun-induced neurotoxic markers measured at 2 h following tabun challenge by the functional observational battery (No. 1–11, 15–35—scored values, No. 12–14, 36–42—values in absolute units). Statistical significance: * *p* < 0.05 (comparison with the control values). Applied abbreviations: RRF, air righting reflex; RRV, air righting reflex from vertical position; *x/M*, average or modus value; ±*s*, standard deviation; A, atropine; n, number of surviving animals.

2 h		Controls		Tabun + A + K203		Tabun + A + Trimedoxime		Tabun + A + K307		Tabun + A + K305		Tabun	
No.	Marker	*x*/*M*	±*s*	*x*/*M*	±*s*	*x*/*M*	±*s*	*x*/*M*	±*s*	*x*/*M*	±*s*	*x*/*M*	±*s*
1	Posture	1.00		4.00 *		3.00		3.00		4.00 *		7.00 *	
2	Catch Difficulty	2.00		2.00		2.00		2.00		1.00 *		1.00 *	
3	Ease of Handling	2.00		2.00		2.00		2.00		2.00		1.00 *	
4	Muscular Tonus	0.00		0.00		0.00		0.00		0.00		-2.00 *	
5	Lacrimation	0.00		0.00		0.00		0.00		0.00		0.00	
6	Palpebral Closure	1.00		1.00		1.00		1.00		1.00		1.00	
7	Endo/Exophtalmus	0.00		0.00		0.00		0.00		0.00		1.00 *	
8	Fur Abnormalities	0.00		2.00 *		0.00		0.00		0.00		7.00 *	
9	Skin Abnormalities	0.00		1.00 *		0.00		0.00		0.00		1.00 *	
10	Salivation	0.00		0.00		0.00		0.00		2.00 *		2.00 *	
11	Nose Secretion	0.00		0.00		0.00		0.00		0.00		3.00 *	
12	Rearing	7.88	8.41	2.50	5.24	4.38	5.42	1.13 *	2.47	0.13 *	0.35	0.00 *	0.00
13	Urination	0.00		0.00		0.00		0.00		0.00		0.00	
14	Defecation	0.00		0.00		0.00		0.00		0.00		0.00	
15	Hyperkinesis	0.00		0.00		0.00		0.00		6.00 *		7.00 *	
16	Tremors	0.00		0.00		0.00		0.00		5.00 *		5.00 *	
17	Clonic Movements	0.00		0.00		0.00		0.00		1.00 *		2.00 *	
18	Tonic Movements	0.00		0.00		0.00		0.00		5.00 *		5.00 *	
19	Gait	0.00		7.00 *		0.00		1.00 *		7.00 *		7.00 *	
20	Ataxia	0.00		2.00 *		0.00		1.00 *		2.00 *		2.00 *	
21	Gait Score	1.00		1.00		1.00		1.00		2.00 *		2.00 *	
22	Mobility Score	1.00		1.00		1.00		1.00		4.00 *		4.00 *	
23	Activity	4.00		4.00		4.00		4.00		1.00 *		1.00 *	
24	Tension	0.00		0.00		0.00		0.00		0.00		2.00 *	
25	Vocalisation	0.00		0.00		0.00		0.00		0.00		0.00	
26	Stereotypy	0.00		0.00		0.00		0.00		0.00		0.00	
27	Bizzare Behavior	0.00		0.00		0.00		0.00		0.00		5.00 *	
28	Approach Response	2.00		2.00		2.00		2.00		1.00 *		1.00 *	
29	Touch Response	2.00		2.00		2.00		2.00		1.00 *		1.00 *	
30	Click Response	2.00		2.00		2.00		2.00		2.00		1.00 *	
31	Tail-Pinch Response	2.00		2.00		2.00		2.00		1.00 *		1.00 *	
32	Pupil Size	0.00		−2.00 *		−2.00 *		−2.00 *		−2.00 *		−2.00 *	
33	Pupil Response	1.00		0.00 *		0.00 *		0.00 *		0.00 *		0.00 *	
34	RRF	1.00		4.00 *		2.00 *		2.00 *		2.00 *		4.00 *	
35	RRV	1.00		1.00		1.00		3.00 *		4.00 *		4.00 *	
36	Landing Foot Splay (mm)	9.41	2.51	7.86	2.10	8.05	2.21	7.15	2.40	4.76 *	3.75	3.51 *	3.15
37	Forelimb Grip Strength (kg)	8.54	2.32	5.54	4.26	6.00	3.49	5.17	3.49	1.39 *	2.98	0.94 *	1.10
38	Hindlimb Grip Strength (Kg)	2.08	0.50	0.87 *	0.55	1.04 *	0.47	1.03 *	0.89	0.34 *	0.32	0.47 *	0.43
39	Grip Strength of all Limbs (kg)	13.89	2.25	7.40 *	4.58	11.68	6.51	10.50	6.59	3.40 *	4.38	2.41 *	2.10
40	Vertical Activity	53.88	32.70	5.13 *	4.82	3.38 *	4.50	13.13 *	3.52	0.75 *	2.12	0.00 *	0.00
41	Horizontal Activity	316.13	120.85	249.00	45.39	327.13	219.49	278.63	216.51	136.63 *	154.11	1.63 *	4.60
42	Total Motor Activity	370.00	147.64	254.13	45.80	330.50	218.87	291.75	238.41	137.38 *	155.60	1.63 *	4.60
		**n = 8**		**n = 7**		**n = 8**		**n = 8**		**n = 6**		**n = 3**	

**Table 2:** The values of tabun-induced neurotoxic markers measured at 24 h following tabun challenge by the Functional observational battery (No. 1–11, 15–35–scored values, No. 12–14, 36–42—values in absolute units). Statistical significance: * *p* < 0.05 (comparison with the control values). Applied abbreviations: RRF, air righting reflex; RRV, air righting reflex from vertical position; *x/M*, average or modus value; ±*s*, standard deviation; A, atropine; n, number of surviving animals.

24 h		Controls		Tabun + A + K203		Tabun + A + Trimedoxime		Tabun + A + K307		Tabun + A + K305		Tabun	
No.	Marker	*x/M*	±*s*	*x/M*	±*s*	*x/M*	±*s*	*x/M*	±*s*	*x/M*	±*s*	*x/M*	±*s*
1	Posture	1.00		1.00		1.00		1.00		7.00 *		7.00 *	
2	Catch Difficulty	2.00		2.00		2.00		2.00		1.00 *		1.00 *	
3	Ease of Handling	2.00		2.00		2.00		1.00 *		1.00 *		1.00 *	
4	Muscular Tonus	0.00		0.00		0.00		0.00		−2.00 *		−2.00 *	
5	Lacrimation	0.00		0.00		0.00		0.00		4.00 *		4.00 *	
6	Palpebral Closure	1.00		1.00		1.00		1.00		5.00 *		5.00 *	
7	Endo/Exophtalmus	0.00		0.00		0.00		0.00		−1.00 *		−1.00 *	
8	Fur Abnormalities	0.00		0.00		0.00		0.00		7.00 *		7.00 *	
9	Skin Abnormalities	0.00		0.00		0.00		0.00		4.00 *		4.00 *	
10	Salivation	0.00		0.00		0.00		0.00		2.00 *		2.00 *	
11	Nose Secretion	0.00		0.00		0.00		1.00 *		3.00 *		3.00 *	
12	Rearing	0.00		0.00		0.00		0.00		0.00		0.00	
13	Urination	0.63	1.19	3.13	3.72	0.38	1.06	3.00	4.04	0.25	0.46	0.13	0.35
14	Defecation	0.00		0.00		0.00		0.00		0.00		0.00	
15	Hyperkinesis	0.00		0.00		0.00		0.00		7.00 *		7.00 *	
16	Tremors	0.00		0.00		0.00		0.00		5.00 *		5.00 *	
17	Clonic Movements	0.00		0.00		0.00		0.00		2.00 *		2.00 *	
18	Tonic Movements	0.00		0.00		0.00		0.00		5.00 *		5.00 *	
19	Gait	0.00		0.00		0.00		0.00		7.00 *		7.00 *	
20	Ataxia	0.00		0.00		0.00		0.00		2.00 *		2.00 *	
21	Gait Score	0.00		0.00		0.00		0.00		2.00 *		2.00 *	
22	Mobility Score	1.00		1.00		1.00		1.00		4.00 *		4.00 *	
23	Activity	4.00		4.00		4.00		4.00		1.00 *		1.00 *	
24	Tension	0.00		0.00		0.00		0.00		2.00 *		2.00 *	
25	Vocalisation	0.00		0.00		0.00		0.00		3.00 *		3.00 *	
26	Stereotypy	0.00		0.00		0.00		0.00		5.00 *		5.00 *	
27	Bizzare Behavior	0.00		0.00		0.00		0.00		5.00 *		5.00 *	
28	Approach Response	2.00		2.00		2.00		1.00 *		1.00 *		1.00 *	
29	Touch Response	2.00		2.00		2.00		2.00		1.00 *		1.00 *	
30	Click Response	2.00		2.00		2.00		2.00		1.00 *		1.00 *	
31	Tail-Pinch Response	2.00		2.00		1.00 *		1.00 *		1.00 *		1.00 *	
32	Pupil Size	0.00		0.00		0.00		0.00		−2.00 *		−2.00 *	
33	Pupil Response	1.00		1.00		1.00		1.00		0.00 *		0.00 *	
34	RRF	1.00		1.00		1.00		1.00		4.00 *		4.00 *	
35	RRV	1.00		1.00		2.00 *		1.00		4.00 *		4.00 *	
36	Landing Foot Splay (mm)	10.20	1.39	5.89 *	4.14	5.83 *	3.66	6.22 *	4.49	3.80 *	4.09	1.08 *	2.06
37	Forelimb Grip Strength (kg)	8.78	2.35	6.68	5.16	7.06	4.55	5.72	4.37	1.94 *	2.90	0.33 *	0.61
38	Hindlimb Grip Strength (kg)	2.54	0.41	1.40 *	1.01	1.78	1.25	1.43 *	1.08	0.54 *	0.71	0.24 *	0.49
39	Grip Strength of all Limbs (kg)	12.67	2.14	9.29	5.99	10.23	6.51	11.80	9.68	4.82 *	5.97	1.41 *	2.61
40	Vertical Activity	40.38	29.81	19.38	27.54	11.00 *	12.36	5.75 *	6.61	0.63 *	0.77	0.00 *	0.00
41	Horizontal Activity	224.63	91.36	94.25 *	85.62	99.25 *	101.98	94.63 *	119.90	23.63 *	47.51	2.38 *	5.29
42	Total Motor Activity	265.00	109.52	113.63 *	111.23	110.25 *	113.60	100.38 *	125.72	24.25 *	49.20	2.38 *	5.29
		**n = 8**		**n = 6**		**n = 6**		**n = 6**		**n = 4**		**n = 3**	

**Table 3:** Percentage of reactivation of tabun-inhibited AChE by oximes in rat brain in vivo.

Treatment	AChE Activity (μ kat/kg)
	Brain
Controls	123.1 ± 3.86
Tabun	18.05 ± 3.59 ^a,^*
Tabun + atropine + K203 (% reactivation ^b^)	27.22 ± 3.55 * (8.7)
Tabun + atropine + trimedoxime (% reactivation)	32.59 ± 6.02 * (13.8)
Tabun + atropine + K305 (% reactivation)	23.09 ± 6.93 (4.8)
Tabun + atropine + K307 (% reactivation)	28.99 ± 3.24 * (10.4)

^a^ Means ± S.E.M., N = 3 − 6; ^b^ % reactivation was determined using the AChE activity values: {1 − [((controls) − (tabun+oxime+atropine))/((controls) − (tabun))]} × 100; * significantly different from the tabun group at the level of *p* < 0.05.

**Table 4:** Score of tabun-induced histopathological damage in rat brain and its modulation by different therapeutic approaches.

	AMG					CRBL					CTX					HIPP					HYPOTH					PIRI					TH				
Score scale	0	1	2	3	4	0	1	2	3	4	0	1	2	3	4	0	1	2	3	4	0	1	2	3	4	0	1	2	3	4	0	1	2	3	4
Controls	4	4	0	0	0	8	0	0	0	0	8	0	0	0	0	8	0	0	0	0	7	1	0	0	0	7	1	0	0	0	8	0	0	0	0
Tabun	0	0	1	0	2	**0**	**1**	**1**	**1**	**0**	**0**	**0**	**0**	**0**	**3**	**0**	**1**	**0**	**2**	**0**	0	1	1	1	0	**0**	**0**	**0**	**1**	**2**	**0**	**1**	**0**	**0**	**2**
Tabun + A + K203	5	0	0	1	0	3	3	0	0	0	***5***	***0***	***0***	***1***	***0***	4	1	1	0	0	3	3	0	0	0	5	0	0	1	0	4	1	1	0	0
Tabun + A + TRI	***5***	***1***	***0***	***0***	***0***	***6***	***0***	***0***	***0***	***0***	***6***	***0***	***0***	***0***	***0***	4	2	0	0	0	5	1	0	0	0	***6***	***0***	***0***	***0***	***0***	***6***	***0***	***0***	***0***	***0***
Tabun + A + K305	0	0	1	0	3	1	2	0	1	0	**0**	**2**	**0**	**0**	**2**	1	1	0	1	1	1	2	0	1	0	1	0	0	1	2	**0**	**1**	**1**	**1**	**1**
Tabun + A + K307	1	2	2	1	0	5	1	0	0	0	1	4	0	1	0	1	4	0	1	0	1	2	1	2	0	1	2	2	0	1	2	1	2	1	0

Each number represents a number of animals suffering from particular damage score in the group. **Bold**: statistically significant compared to control group (*p* ≤ 0.05). ***Bold Italic***: statistically significant compared to tabun-poisoned group (*p* ≤ 0.05). Abbreviations: A—atropine, AMG—amygdaloid body, CRBL—cerebellum, CTX—cortex, HIPP—hippocampus, HYPOTH—hypothalamus, PIRI—piriform cortex, TH—thalamus, TRI—trimedoxime.

**Table 5:** Score of tabun-induced degeneration (Fluoro-Jade^®^ C positivity) in rat brain and its modulation by different therapeutic approaches.

	AMG					CRBL					CTX					HIPP					HYPOTH					PIRI					TH				
Score scale	0	1	2	3	4	0	1	2	3	4	0	1	2	3	4	0	1	2	3	4	0	1	2	3	4	0	1	2	3	4	0	1	2	3	4
Controls	8	0	0	0	0	8	0	0	0	0	8	0	0	0	0	8	0	0	0	0	7	1	0	0	0	8	0	0	0	0	8	0	0	0	0
Tabun	**0**	**1**	**0**	**0**	**2**	2	0	1	0	0	**0**	**1**	**0**	**0**	**2**	1	0	0	0	2	1	2	0	0	0	**0**	**1**	**0**	**0**	**2**	1	0	0	1	1
Tabun + A + K203	4	0	1	0	1	6	0	0	0	0	5	0	0	0	1	4	1	0	0	1	3	2	0	1	0	5	0	0	0	1	4	0	1	0	1
Tabun + A + TRI	***6***	***0***	***0***	***0***	***0***	6	0	0	0	0	4	2	0	0	0	4	2	0	0	0	1	5	0	0	0	5	1	0	0	0	3	1	2	0	0
Tabun + A + K305	1	0	0	2	1	4	0	0	0	0	1	0	0	1	2	**0**	**1**	**1**	**0**	**2**	1	3	0	0	0	1	0	0	1	2	**0**	**1**	**0**	**2**	**1**
Tabun + A + K307	3	2	0	0	1	5	1	0	0	0	4	0	1	0	1	1	4	0	0	1	3	3	0	0	0	1	2	0	2	1	3	1	1	0	1

Each number represents a number of animals suffering from particular damage score in the group. **Bold**: statistically significant compared to control group (*p* ≤ 0.05). ***Bold Italic***: statistically significant compared to tabun-poisoned group (*p* ≤ 0.05). Abbreviations: A—atropine, AMG—amygdaloid body, CRBL—cerebellum, CTX—cortex, HIPP—hippocampus, HYPOTH—hypothalamus, PIRI—piriform cortex, TH—thalamus, TRI—trimedoxime.

**Table 6:** Functional observational battery (FOB).

Marker	Scored Values Only									
	−2	−1	0	1	2	3	4	5	6	7
Posture				*sitting or standing*	*rearing*	*asleep*	flattened	lying on side	crouched over	head bobbing
Catch Difficulty				passive	*normal*	defense	flight	escape	aggrression	
Ease of Handling				very easy	*easy*	moderately difficult	difficult			
Muscular Tonus	atonia	hypotonia	*normal*	hypertonia	rigidity	fasciculations				
Lacrimation			*none*	slight	severe	crusta	coloured crusta			
Palpebral Closure				*open*	slightly	half-way	completely	ptosis		
Endo-Exophthalmus		endo	*normal*	exo						
Piloerection			*no*	yes						
Skin Abnormalities			*normal*	pale	erythema	cyanosis	pigmented	cold	injury	
Salivation			*none*	sllight	severe					
Nose Secretion			*none*	slight	severe	coloured				
Clonic Movements			*normal*	repetitive	nonrhythmic	mild tremors	severe tremors	myoclonic	clonic	
Tonic Movements			*normal*	contraction of extensors	opisthotonus	emprostho- tonus	explosive jumps	tonic convulsions		
Gait			*normal*	ataxia	overcompen- sation of hindlimbs movements	feet point outwards from body	forelimbs are extended	walks on tiptoes	hunched body	body is flattened against surface
Gait Score				*normal*	slightly impaired	somewhat impaired	totally impaired			
Mobility Score				*normal*	slightly impaired	somewhat impaired	totally impaired			
Arousal (Level of Unprovoked Activity)				very low	sporadic	reduced	*normal*	enhanced	permanent	
Tension			*none*	partial (ears)	stupor					
Stereotypy			*none*	head weaving	body weaving	grooming	circling	others		
Bizarre Behavior			*none*	head	body	self-mutilation	abnormal movements	others		
Approach Response				no reaction	*normal*	slow reaction	energetic reaction	exaggerated reaction		
Touch Response				no reaction	*normal*	slow reaction	energetic reaction	exaggerated reaction		
Click Response				no reaction	*normal*	slow reaction	energetic reaction	exaggerated reaction		
Tail-Pinch Response				no reaction	*normal*	slow reaction	energetic reaction	exaggerated reaction		
Pupil Size		miosis	*normal*	mydriasis						
Pupil Response			no reaction	*normal reaction*						
Righting Reflex				*normal*	slightly uncoordin.	lands on side	lands on back			

## References

[1] Bajgar J. (2004). Organophosphate/nerve agent poisoning: Mechanism of action, diagnosis, prophylaxis, and treatment. Adv. Clin. Chem..

[2] Delfino R.T., Ribeiro T.S., Figueroa-Villar J.D. (2009). Organophosphorus compounds as chemical warfare agents: A review. J. Braz. Chem. Soc..

[3] Colovic M.B., Krstic D.Z., Lazarevic-Pasti T.D., Bondzic A.M., Vasic V.M. (2013). Acetylcholinesterase Inhibitors: Pharmacology and Toxicology. Curr. Neuropharmacol..

[4] Jokanovic M., Prostran M. (2009). Pyridinium oximes as cholinesterase reactivators. Structure-activity relationship and efficacy in the treatment of poisoning with organophosphorus compounds. Curr. Med. Chem..

[5] Kassa J., Musilek K., Zdarova Karasova J., Kuca K., Bajgar J. (2012). Two possibilities how to increase the efficacy of antidotal treatment of nerve agent poisonings. Mini-Rev. Med. Chem..

[6] Cabal J., Bajgar J. (1999). Tabun—Reappearance 50 years later. Chem. Listy.

[7] Ekström F., Akfur C., Tunemalm A.K., Lundberg S. (2006). Structural changes of phenylalanine 338 and histidine 447 revealed by the crystal structures of tabun-inhibited murine acetylcholinesterase. Biochemistry.

[8] Hoffman A., Eisenkraft A., Finkelstein A., Schein O., Rotman E., Dushnitsky T. (2007). A decade after the Tokyo sarin attack: A review of neurological follow-up of the victims. Mil. Med..

[9] Yamasue H., Abe O., Kasai K., Suga M., Iwanami A., Yamada A., Tochigi M., Ohtani T., Rogers M.A., Sasaki T. (2007). Human brain structural changes related to acute single exposure to sarin. Ann. Neurol..

[10] Cassel G., Karlsson L., Waara L., Wee Ang K., Goransson-Nyberg A. (1997). Pharmacokinetics and effects of HI-6 in blood and brain of soman-intoxicated rats: A microdialysis study. Eur. J. Pharmacol..

[11] Sakurada K., Matsubara K., Shimizu K., Shiono H., Seto Y., Tsuge K., Yoshino M., Sakai I., Mukoyama H., Takatori E. (2003). Pralidoxime iodide (2-PAM) penetrates across the blood-brain barrier. Neurochem. Res..

[12] Lorke D.E., Kalasz H., Petroianu G.A., Tekes K. (2008). Entry of oximes into the brain: A review. Curr. Med. Chem..

[13] Jokanovic M. (2012). Structure-activity relationship and efficacy of pyridinium oximes in the treatment of poisoning with organophosphorus compounds: A review of recent data. Curr. Top. Med. Chem..

[14] Wilhelm C.M., Snider T.H., Babin M.C., Jett D.A., Platoff G.E., Yeung D.T. (2014). A comprehensive evaluation of the efficacy of leading oxime therapies in guinea pigs exposed to organophosphorus chemical warfare agents or pesticides. Toxicol. Appl. Pharmacol..

[15] Sharma R., Gupta B., Singh N., Acharya J.R., Musilek K., Kuca K., Ghosh K.K. (2015). Development and structural modifications of cholinesterase reactivators against chemical warfare agents in last decade: A review. Mini-Rev. Med. Chem..

[16] Kassa J., Karasova J., Musilek K., Kuca K. (2008). An evaluation of therapeutic and reactivating effects of newly developed oximes (K156, K203) with commonly used oximes (obidoxime, trimedoxime, HI-6) in tabun-poisoned rats and mice. Toxicology.

[17] Winter M., Wille T., Musilek K., Kuca K., Thiermann H., Worek F. (2016). Investigation of the reactivation kinetics of a large series of bispyridinium oximes with organophosphate-inhibited human acetylcholinesterase. Toxicol. Lett..

[18] Bajgar J., Jun D., Kuca K., Bartosova L., Fusek J. (2007). Cholinesterase reactivators: The fate and effects in the organism poisoned with organophosphates/nerve agents. Curr. Drug Metab..

[19] Dolgin E. (2013). Syrian gas attack reinforces need for better anti-sarin drugs. Nat. Med..

[20] Pita R., Domingo J. (2014). The use of chemical weapons in the Syrian conflict. Toxics.

[21] Shih T.M., Duniho S.M., McDonough J.H. (2003). Control of NA-induced seizures is critical for neuroprotection and survival. Toxicol. Appl. Pharmacol..

[22] Marrs T.C., Marrs T.C., Maynard R.L., Sidell F.R. (2007). Toxicology of Organophosphate Nerve Agents. Chemical Warfare Agents: Toxicology and Treatment.

[23] Chen Y. (2012). Organophosphate-induced brain damage: Mechanisms, neuropsychiatric and neurological consequences, and potential therapeutic strategies. NeuroToxicology.

[24] Weissman B.A., Raveh L. (2008). Therapy against organophosphate poisoning: The importance of anticholinergic drugs with antiglutamatergic properties. Toxicol. Appl. Pharmacol..

[25] Kassa J., Kunesova G. (2006). Comparison of the neuroprotective effects of the newly developed oximes (K027, K048) with trimedoxime in tabun-poisoned rats. J. Appl. Biomed..

[26] McDonough J.H., Zoeffel L.D., McMonagle J., Copeland T.L., Smith C.D., Shih T.-M. (2000). Anticonvulsant treatment of nerve agent seizures: Anticholinergics versus diazepam in soman-intoxicated guinea-pigs. Epilepsy Res..

[27] Antonijevic B., Stojiljkovic P. (2007). Unequal efficacy of pyridinium oximes in acute organophosphate poisoning. Clin. Med. Res..

[28] Nurulain S.M. (2011). Efficacious oxime for organophosphorus poisoning: A minireview. Trop. J. Pharm. Res..

[29] Kassa J., Krejcova G. (2003). Neuroprotective effects of currently used antidotes in tabun-poisoned rats. Pharmacol. Toxicol..

[30] Kassa J., Karasova J., Vasina L., Bajgar J., Kuca K., Musilek K. (2009). A comparison of neuroprotective efficacy of newly developed oximes (K203, K206) and commonly used oximes (obidoxime, HI-6) in tabun-poisoned rats. Drug Chem. Toxicol..

[31] Worek F., Widmann R., Knopff O., Szinicz L. (1998). Reactivating potency of obidoxime, pralidoxime, HI-6 and HLö-7 in human erythrocyte acetylcholinesterase inhibited by highly toxic organophosphorus compounds. Arch. Toxicol..

[32] Zdarova Karasova J., Pohanka M., Musilek K., Zemek F., Kuca K. (2010). Passive diffusion of acetylcholinesterase oxime reactivators through the blood-brain barrier: Influence of molecular structure. Toxicol. In Vitro.

[33] Cabal J., Kuca K., Kassa J. (2004). Specification of the structure of oximes able to reactivate tabun-inhibited acetylcholinesterase. Basic Clin. Pharmacol. Toxicol..

[34] Kuca K., Jun D., Musilek K. (2006). Structural requirements of acetylcholinesterase reactivators. Mini Rev. Med. Chem..

[35] Musilek K., Kuca K., Jun D., Dolezal M. (2007). Progress in synthesis of new acetylcholinesterase reactivators during the period 1990–2004. Curr. Org. Chem..

[36] Biljana A., Slavica V., Cupic V. (2012). Protective effect of HI-6 and trimedoxime combination in mice acutely poisoned with tabun, dichlorvos and heptenophos. Acta Vet. Beogr..

[37] Kassa J., Sepsova V., Tumova M., Horova A., Musilek K. (2015). A comparison of the reactivating and therapeutic efficacy of two newly developed oximes (K727, K733) with oxime K203 and trimedoxime in tabun-poisoned rats and mice. Basic Clin. Pharmacol. Toxicol..

[38] Kadar T., Shapira S., Cohen G., Sahar R., Alkalay D., Raveh L. (1995). Sarin-induced neuropathology in rats. Hum. Exp. Toxicol..

[39] Jun D., Kuca K., Stodulka P., Koleckar V., Dolezal B., Simon P., Veverka M. (2007). HPLC analysis of HI-6 dichloride and dimethanesulfonate - antidotes against nerve agents and organophosphorus pesticides. Anal. Lett..

[40] Kassa J., Sepsova V., Horova A., Musilek K. (2017). A comparison of the reactivating and therapeutic efficacy of two novel bispyridinium oximes (K305, K307) with the oxime K203 and trimedoxime in tabun-poisoned rats and mice. J. Appl. Biomed..

[41] Moser V.C., Tilson H., McPhail R.C., Becking G.C., Cuomo V., Frantik E., Kulig B.M., Winneke G. (1997). The IPCS collaborative study on neurobehavioral screening methods: II. Protocol design and testing procedures. NeuroToxicology.

[42] Ellman G.L., Courtney D.K., Andres V., Feartherstone R.M. (1961). A new and rapid colorimetric determination of acetylcholinesterase activity. Biochem. Pharmacol..

[43] Clement J.G., Hansen A.S., Boulet C.A. (1992). Efficacy of HLö-7 and pyrimidoxime as antidotes of nerve agent poisoning in mice. Arch. Toxicol..

[44] Paxinos G., Watson C. (2006). The Rat Brain in Stereotactic Coordinates.

